# Mortality impacts of the coronavirus disease (COVID-19) outbreak by sex and age: rapid mortality surveillance system, Italy, 1 February to 18 April 2020

**DOI:** 10.2807/1560-7917.ES.2020.25.19.2000620

**Published:** 2020-05-14

**Authors:** Paola Michelozzi, Francesca de’Donato, Matteo Scortichini, Manuela De Sario, Fiammetta Noccioli, Pasqualino Rossi, Marina Davoli

**Affiliations:** 1 Department of Epidemiology, Lazio Regional Health Service, ASL Roma 1, Rome, Italy; 2 Health Prevention Directorate, Italian Ministry of Health, Rome, Italy

**Keywords:** COVID-19, surveillance system, mortality, demographic factors, vulnerable populations

## Abstract

Data from the rapid mortality surveillance system in 19 major Italian cities were used to carry out a timely assessment of the health impact of the COVID-19 epidemic. By 18 April, a + 45% excess in mortality was observed, with a higher impact in the north of the country (+ 76%). The excess was greatest among men, with an increasing trend by age. Surveillance data can be used to evaluate the lockdown and re-opening phases.

Italy has been one of the countries worst hit by coronavirus disease (COVID-19), with over 185,000 cases and around 80% registered in north of the country [1]. Numbers in the initial phase of the outbreak in Italy seem to suggest a greater severity of the disease, with a higher case fatality rate (CFR) than previously observed in China (7.2% vs 2.3%) [2].

The aim of the study was to estimate the excess in total mortality by age and sex during the epidemic in Italian cities.

## The rapid mortality surveillance system in Italy

Since 2004, Italy has had a rapid mortality surveillance system (SiSMG) for real-time monitoring of daily deaths in major Italian cities and allows routine evaluation of the health impact of extreme events and influenza epidemics [3,4]. This surveillance system was a valuable tool for an early evaluation of the direct or indirect impact of COVID-19 on health. It is a standardised surveillance system capable of detecting variations in total mortality in the entire population rather than only on the infected cases, and it does not depend on a specific case definition (i.e. COVID-19-related deaths).

Briefly, SiSMG is based on an ad hoc daily flow of mortality data (resident population by age and sex) from local Municipal Registry Offices to the Department of Epidemiology, Lazio Regional Health Authority - ASL Roma 1 (DEPLAZIO) which manages the system on behalf of the Ministry of Health [5,6]. The standardised methodology to evaluate excess mortality typically used in the Italian national surveillance system was considered when estimating the excess related to COVID-19 [3,5-7]. Specifically, the excess was defined as the difference between observed and baseline daily mortality (mean daily value by week and day of the week in the past 5 years). In this report, we show results for a subgroup of 19 cities, representative of almost all Italian regions, with timely updates of data, corresponding to 9 million residents (14% of the Italian population).

## Excess total mortality during the coronavirus disease epidemic 

**Figure 1:**
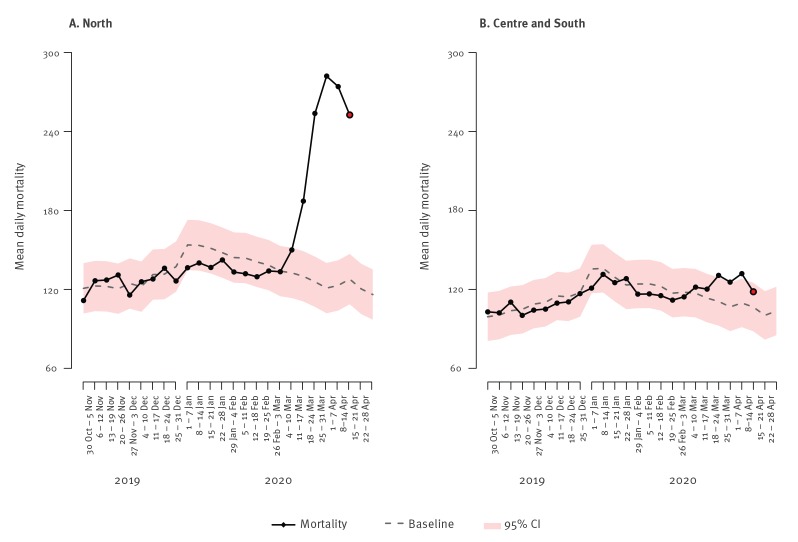
Weekly trend in mean daily observed and expected mortality in northern^a^ and central-southern^b^ cities during the COVID-19 outbreak, Italy, November 2019–April 2020

CI: confidence interval; COVID-19: coronavirus disease; SiSMG: Italian rapid mortality surveillance system.

^a^ North (Aosta, Bolzano, Turin, Milan, Brescia, Verona, Venice, Trieste, Trento, Genoa, Bologna).

^b^ Centre and south (Perugia, Civitavecchia, Rome, Frosinone, Bari, Potenza, Palermo, Messina).

Figure 1 shows the weekly trend in observed and expected (baseline) mortality in northern vs central and southern cities of Italy included in SiSMG. Shaded areas represent the confidence bands calculated as the estimated baseline +/− 1.96 × standard deviation of the series of observed deaths contributing to the baseline. From the first week of March, a steep rise in mortality was observed in cities in northern Italy (Figure 1). From the start of the COVID-19 epidemic until 18 April, an overall 4,805 (+ 45%) excess deaths were observed in Italian cities, with a significantly higher excess in cities in the north (+ 76%, +4,295 deaths) compared with the centre and south (+ 10%, + 510 deaths). 

Black line: observed mortality; dashed line: expected mortality; pink shading: 95% CI.

Data source: SiSMG surveillance system, Ministry of Health Italy. Originally published in [6].

City-specific mortality data showed the highest excess in the cities in the north, especially Brescia (+ 197%) and Milan (+ 103%), Lombardy, Genoa, Liguria (+ 84%), Turin, Piedmont (+ 57%), Verona, Veneto (+ 40%) and Bologna and Emilia-Romagna (+ 47%). In contrast, the south and central cities recorded a more contained (+ 7% in Rome, + 20% in Messina) or no excess.

**Figure 2:**
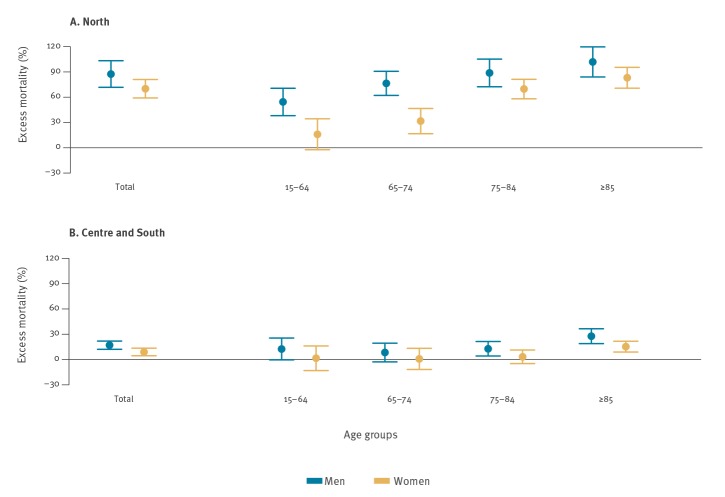
Excess mortality by sex and age groups (all ages, 15–64, 65–74, 75–84, ≥ 85 years) in northern^a^ vs central and southern^b^ cities, COVID-19 outbreak, Italy, starting date^c^–18 April 2020

COVID-19: coronavirus disease; SiSMG: Italian rapid mortality surveillance system.

^a^ North (Aosta, Bolzano, Turin, Milan, Brescia, Verona, Venice, Trieste, Trento, Genoa, Bologna).

^b^ Centre and south (Perugia, Civitavecchia, Rome, Frosinone, Bari, Potenza, Palermo, Messina).

^c^ Starting dates: Palermo, Turin, Venice: 25 February; Brescia: 26 February; Bari: 27 February; Bologna, Civitavecchia, Rome: 29 February; Genoa, Perugia, Trieste, Verona: 1 March; Potenza, Trento: 3 March; Frosinone: 4 March; Aosta: 5 March; Messina: 7 March [1].

Figure 2 shows excess mortality by sex and age groups among cities in the north and in the centre and south of Italy caused by the COVID-19 outbreak up until 3 April 2020. Overall, the excess in mortality was higher among men than among women in cities in the north vs the centre and south of Italy (men:+ 87% and + 70% and women: + 17% and + 9%, respectively), with an increasing trend by age. The greatest excess in the north was among elderly men (+ 76% in 65–74 year-olds, + 89% in 75–84 year-olds and + 102% in those 85 years and older). In central and southern Italy, the excess in mortality among men was lower, with a statistically significant excess only among elderly men: + 13% and + 28%, respectively, in the 75–84 years and ≥ 85 years age group.

Data source: SiSMG surveillance system, Ministry of Health Italy [5,6].

## Discussion

Our results confirm that the epidemic had the strongest impact in the most affected areas of the North. National COVID-19 data show that the Lombardy region alone accounted for 40% of cases in Italy, followed by the neighbouring regions of Emilia-Romagna (12%), Piedmont (12%) and Veneto (9%) [1]. It should be considered that these four northern regions accounted for ca 70% of national trade and economic activity [8], which could have accelerated and amplified the spread of infection to and within this area of Italy. The regions of central and southern Italy registered nearly a week’s delay in the outbreak and cases are to date much lower, with ca 20% of total cases [1]. Central-southern cities were comparatively less affected, possibly because containment measures were put in place before the emergency stage was reached.

When considering the impact of COVID-19 mortality, we should take into account the low mortality of the 2019/20 winter, below the baseline from October to the end of February, which was attributable to the mild intensity of the influenza season and a warmer than average temperatures as shown in the latest SiSMG report [7]. This phenomenon of lower mortality may have led to a greater number of vulnerable individuals exposed to the COVID-19 epidemic, thus amplifying the impact on mortality in our country.

Prevalence of chronic diseases is an important risk factor to take into account when analysing excess mortality related to COVID-19. Previous studies have shown that both disease severity and death risk are higher in patients with co-existing illnesses [9,10]. Recent data from Italy show that ca 60% of COVID-19 deaths occurred in people with three or more comorbidities, mainly hypertension (69%), type-2 diabetes (32%), chronic renal failure (21%) and ischaemic heart disease (27%) [11]. In Italian cities, the higher mortality impact in men confirmed findings from a previous study in Italy [2]. Furthermore, experimental studies suggest that men are more susceptible to respiratory viral infections because of hormonal and epigenetic mechanisms involving innate immunity [12]. This has also been shown in animal studies for severe acute respiratory syndrome (SARS) coronavirus [13]. Another potential risk factor reported in the literature is smoking. Firstly, smoking is detrimental to the immune system and its responsiveness to infections and, specifically, it is able to upregulate the ACE-2 receptors in the airways, the key for SARS-CoV-2 virus entrance into the epithelial cells [10]. A recent review showed that smoking is most probably associated with a negative progression and adverse outcomes of COVID-19 [10,14]. The higher prevalence of male smokers for all ages in Italy, especially among the elderly [15], may explain their higher predisposition to COVID-19.

Furthermore, the potential role of air pollution has been as a possible factor influencing COVID-19 transmission and an effect modifier of the number of cases [16,17]. Populations with a chronic exposure to high levels of air pollution, such as in the north of Italy [18], may have a greater predisposition to develop respiratory symptoms, which may make them more susceptible to COVID-19. However, evidence on the direct and indirect role of air pollution is to date limited and the causal pathways and the differential risk attributable to this in different geographical areas need to be further investigated.

Considering our findings in the European context, the latest EuroMOMO bulletin shows a steep increase in excess all-cause mortality in several European countries, mostly in the age groups ≥ 65 and 15–64 years [19]. A comparison of official COVID-19 deaths in Europe and the United States suggests that adults younger than 65 years had a lower death risk than elderly people, with high geographical variability, and that more than 99% of deaths occurred among people with previous illnesses [20]. The different risk factors associated with COVID-19 predisposition and severity need to be taken into account when assessing the geographical heterogeneity in the risk of COVID-19-related deaths in the different population subgroups.

Since the beginning of the Italian epidemic, efforts have been made to estimate the impact, which could possibly be greater than indicated by official data [1] because of a delay in death ascertainment and lack of specificity of COVID-19-related deaths. Despite these methodological challenges, rapid surveillance systems can help provide timely updates of the overall impact at population level and reference data for evaluation studies on lockdown measures during the epidemic. They can also help guide the re-opening phase in different areas.

## References

[1] COVID-19 dati-regioni. [COVID-19 Italy: regional data digital repository]. Rome: Department for Civil Protection. [Accessed: 13 Apr2020]. Italian. Available from: https://github.com/pcm-dpc/COVID-19/tree/master/dati-regioni

[2] Onder G Rezza G Brusaferro S Case-fatality rate and characteristics of patients dying in relation to COVID-19 in Italy. JAMA. 2020. 10.1001/jama.2020.4683 32203977

[3] de’Donato FK Leone M Noce D Davoli M Michelozzi P The impact of the February 2012 cold spell on health in Italy using surveillance data. PLoS One. 2013;8(4):e61720. 10.1371/journal.pone.0061720 23637892 PMC3630119

[4] Vestergaard LS Nielsen J Krause TG Espenhain L Tersago K Bustos Sierra N Excess all-cause and influenza-attributable mortality in Europe, December 2016 to February 2017. Euro Surveill. 2017;22(14):30506. 10.2807/1560-7917.ES.2017.22.14.30506 28424146 PMC5388126

[5] Michelozzi P de’ Donato FK Bargagli AM D’Ippoliti D De Sario M Marino C Surveillance of summer mortality and preparedness to reduce the health impact of heat waves in Italy. Int J Environ Res Public Health. 2010;7(5):2256-73. 10.3390/ijerph7052256 20623023 PMC2898048

[6] Ministry of Health. Sistema di sorveglianza della mortalità giornaliera - rapporto settimanale. Settimana 15-21 Aprile. [The weekly mortality bulletin. Week 15-21 April]. Rome: Ministry of Health. [Accessed: 7 May 2020]. Italian. Available from: http://www.salute.gov.it/portale/caldo/SISMG_sintesi_ULTIMO.pdf

[7] Department of Epidemiology Regional Health Service Lazio (DEPLAZIO) on behalf of the Ministry of Health. Mortalità giornaliera (SiSMG) ed analisi della mortalità cumulative nelle città italiane in relazione all’epidemia di Covid-19. Sesto Rapporto 1 Febbraio – 25 Aprile. [Daily mortality report in Italian cities related to the COVID-19 epidemic. Sixth report 1 February–18 April 2020]. Rome: DEPLAZIO. [Accessed: 27 Apr 2020]. Italian. Available from: http://www.deplazio.net/images/stories/SISMG/SISMG_COVID19.pdf

[8] Ministry of Foreign Affairs and International Cooperation. Statistiche import export. Graduatoria delle regioni italiane per valore delle esportazioni/importazioni in base ai dati 2019. [Import export statistics. Ranking of Italian regions by import-export shares based on 2019 data]. Rome: Ministero degli Affari Esteri e della Cooperazione Internazionale. [Accessed: 24 Apr 2020]. Italian. Available from: https://www.esteri.it/mae/it/politica_estera/commercio-internazionale/osservatorio-commercio-internazionale/statistiche-import-export.html

[9] Guan WJ Ni ZY Hu Y Liang WH Ou CQ He JX Clinical characteristics of coronavirus disease 2019 in China. N Engl J Med. 2020;382(18):1708-20. 10.1056/NEJMoa2002032 32109013 PMC7092819

[10] Cai H Sex difference and smoking predisposition in patients with COVID-19. Lancet Respir Med. 2020;8(4):e20. 10.1016/S2213-2600(20)30117-X 32171067 PMC7103991

[11] National Health Institute (ISS). Characteristics of SARS-CoV-2 patients dying in Italy. Report based on available data on April 29th, 2020. Rome: ISS [Accessed: 7 May 2020]. Available from: https://www.epicentro.iss.it/en/coronavirus/bollettino/Report-COVID-2019_29_april_2020.pdf

[12] Kadel S Kovats S Sex hormones regulate innate immune cells and promote sex differences in respiratory virus infection. Front Immunol. 2018;9:1653. 10.3389/fimmu.2018.01653 30079065 PMC6062604

[13] Karlberg J Chong DSY Lai WYY Do men have a higher case fatality rate of severe acute respiratory syndrome than women do? Am J Epidemiol. 2004;159(3):229-31. 10.1093/aje/kwh056 14742282 PMC7110237

[14] Vardavas CI Nikitara K COVID-19 and smoking: A systematic review of the evidence. Tob Induc Dis. 2020;18(March):20. 10.18332/tid/119324 32206052 PMC7083240

[15] National Health Institute (ISS). La sorveglianza Passi d'Argento. I dati per l'Italia: abitudine al fumo. [The Italian behavioural risk factor surveillance system in the elderly population (PASSI d’Argento). Data for Italy: smoking habit.]. Rome: ISS [Accessed: 24 Apr 2020]. Italian. Available from: https://www.epicentro.iss.it/passi-argento/dati/fumo#dati

[16] Su W Wu X Geng X Zhao X Liu Q Liu T The short-term effects of air pollutants on influenza-like illness in Jinan, China. BMC Public Health. 2019;19(1):1319. 10.1186/s12889-019-7607-2 31638933 PMC6805627

[17] Wu X, Nethery RC, Sabath MB, Braun D, Dominici F. Exposure to air pollution and COVID-19 mortality in the United States. medRxiv [Preprint]. 2020 [Accessed: 7 Apr 2020]. 10.1101/2020.04.05.20054502 PMC7673673 33148655

[18] Stafoggia M Bellander T Bucci S Davoli M de Hoogh K De’ Donato F Estimation of daily PM_10_ and PM_2.5_ concentrations in Italy, 2013-2015, using a spatiotemporal land-use random-forest model. Environ Int. 2019;124:170-9. 10.1016/j.envint.2019.01.016 30654325

[19] EuroMOMO. The European mortality monitoring bulletin for week 15, 2020. Copenhagen: EuroMOMO. [Accessed: 7 May 2020]. Available from: https://www.euromomo.eu/bulletins/2020-15

[20] Ioannidis JPA, Axfors C, Contopoulos-Ioannidis DG. Population-level COVID-19 mortality risk for non-elderly individuals overall and for nonelderly individuals without underlying diseases in pandemic epicenters. medRxiv [Preprint]. 2020 [Accessed: 8 Apr 2020]. 10.1101/2020.04.05.20054361 PMC7327471 32846654

